# Influence of Specimen Dimension, Water Immersion Protocol, and Surface Roughness on Water Sorption and Solubility of Resin-Based Restorative Materials

**DOI:** 10.3390/ma17050984

**Published:** 2024-02-21

**Authors:** Eduardo Moreira da Silva, Cristiane Mariote Amaral, Renata Nunes Jardim, Marianna Pires Barbosa, Tiago Braga Rabello

**Affiliations:** 1 Analytical Laboratory of Restorative Biomaterials—LABiom-R, Faculdade de Odontologia, Universidade Federal Fluminense, Niterói 24040-110, Brazil; cristianemariote@id.uff.br (C.M.A.); rnjardim@id.uff.br (R.N.J.); mariannapiresbarbosa@gmail.com (M.P.B.); 2 Faculdade de Odontologia, Universidade Federal do Rio de Janeiro, Rio de Janeiro 21941-902, Brazil; tiagobragarabello@gmail.com

**Keywords:** resin-based restorative materials, water sorption, solubility

## Abstract

The evaluation of water sorption and solubility is pivotal for the development of new resin-based restorative materials with the potential for clinical application. The purpose of the present study was to evaluate the influence of the specimen dimension, water immersion protocol, and surface roughness on the water sorption and solubility of three resin-based restorative materials. Disk-shaped specimens of 15 mm × 1 mm, 10 mm × 1 mm, and 6 mm × 1 mm were produced with a composite resin (Z100), a resin cement (RelyX ARC), and an adhesive system (Single Bond 2—SB2). The specimens were immersed in distilled water according to four protocols: ISO (all the specimens for each group were vertically immersed in 50 mL); IV-10 (the specimens were individually and vertically immersed in 10 mL); IH-10 (the specimens were individually and horizontally immersed in 10 mL); and IH-2 (the specimens were individually and horizontally immersed in 2 mL). The surface roughness (Sa and Sp) was evaluated using an atomic force microscope, and the degree of conversion was determined using FT-IR spectrometry. The specimen dimension and water immersion protocol had no effect on water sorption and solubility. For the three resin-based restorative materials, Sp was higher than Sa. The degree of conversion was not influenced by the specimen dimension. The variations in the specimen dimension and water immersion protocol compared to those determined by ISO 4049 did not prevent the comparison between the values of water sorption and solubility obtained for a given resin-based restorative material.

## 1. Introduction

Regardless of the restorative technique employed in the modern dental clinic, at least one kind of resin-based restorative material, e.g., dental composite, adhesive system, or resin cement, will be used during the restorative procedure [1]. Basically, resin-based restorative materials comprise an organic matrix of methacrylate monomers (Bis-GMA, Bis-EMA, UDMA, TEGDMA, or HEMA), inorganic filler particles, and a silane-coupling agent that bonds the former two phases together [2]. These three phases exert a direct influence on the physical, chemical, and biological behavior of the resin-based restorative materials [3,4,5,6].

When in service in the hostile and wet intra-oral environment, resin-based restorative materials are immediately and constantly exposed to saliva and harsh extrinsic fluids, e.g., low-pH beverages, alcohol, and acids. As a result, water sorption and solubility may be considered as the first step towards the start of the process of degradation that may impair the physicomechanical properties of these restorative materials inside the oral cavity [7]. Additionally, water sorption and solubility are also correlated with residual monomer leaching, which is a matter of concern in terms of biocompatibility [8]. Therefore, investigations of these phenomena are still relevant to increase the knowledge basis regarding the behavior of resin-based restorative materials and for the development of new materials with the potential for clinical application.

The method that is most often used to evaluate the water sorption and solubility of resin-based restorative materials is described in the ISO 4049 standard [9], which establishes that specimens of 15.0 ± 0.1 mm in diameter and 1.0 ± 0.1 mm thick are to be immersed in water at 37 ± 1 °C for 7 d in such a way that they are vertical and have a minimum of 3 mm separation between them. Also, the volume of water for the immersion of each specimen is to be at least 10 mL. Although many earlier studies complied with these rules [10,11,12,13,14,15,16,17,18,19,20,21], others have used specimens with dimensions (mm) such as 20 × 1.5 [22], 12 × 2 [23], 12 × 1 [24], 10 × 4 [25], 10 × 1 [26,27,28,29,30,31,32], 8 × 2 [33], 8 × 1.7 [34], 8 × 1 [35], 7 × 1 [36,37], 6 × 2 [38], 6 × 1 [6,39,40,41,42,43,44,45,46,47], 6 × 0.5 [48], 5 × 2 [49,50], 5 × 1 [51], and 5.8 × 0.8 [52,53]. In addition, some of these studies have also employed modifications in the protocol established by ISO 4049 for immersing the specimens in water, or they have omitted this information. Most probably, these variations in the methods used by the dental scientists can be explained by factors such as the difficulty in building up specimens with a 15 mm diameter without edge defects; the necessity of light-curing larger specimens using overlapping methods, which may lead to inhomogeneous polymerization; and the difficulty in maintaining the specimens in a vertical position during the water immersion step. The variations may also be due to economic factors. According to some authors [54,55], these dissimilarities in methods make it difficult to compare the results obtained in different studies regarding water sorption and solubility for a given resin-based restorative material.

Roughness is the property that expresses the texture of a surface, which is represented by the random deviations from the nominal surface that form its three-dimensional topography [56]. Even when classified as smooth, any surface still presents defects on the micro- and nanoscale. Consequently, when two solid surfaces come into contact, their roughness causes contact to occur only at discrete contact spots in the interface [57]. From this picture, it is reasonable to claim that water, a nanosized molecule, could easily flow through this interface [58].

The primary purpose of the present study was to investigate whether the results of the water sorption and solubility of resin-based restorative materials obtained using specimens with different dimensions and submitted to different water immersion protocols were similar to those obtained using the protocol established in the ISO 4049 standard. The research hypothesis was that the dimension of the specimen and the water immersion protocol would not interfere with the values of water sorption and solubility for each of the three resin-based restorative materials. A secondary objective was to evaluate the nano-roughness of the three resin-based restorative materials and their influence on water sorption and solubility.

## 2. Materials and Methods

### 2.1. Preparation of Specimens

A resin composite, a resin cement, and an adhesive system were used in the present study (Table 1). A total of 60 disk-shaped specimens, 20 (15 mm × 1 mm), 20 (10 × 1 mm), and 20 (6 mm × 1 mm), were built up for each resin-based restorative material. 

The specimens were prepared using aluminum split molds with the dimensions (mm) of each respective group. For the resin cement specimens, equal amounts of base paste and catalyst paste were dispensed on a clean mixing pad and mixed for 20 s according to the manufacturers’ instructions. For adhesive system specimens, the solvent was allowed to evaporate as previously described [42]. Briefly, Adper Single Bond 2 was dispensed into a container on an analytical balance (XP205, Mettler-Toledo, Greifensee, Switzerland), and its mass was recorded until it reached equilibrium. The molds were positioned on a glass slide covered with a polyester strip, and the resin-based restorative materials were carefully inserted into them; they were then covered with another polyester strip and another glass slide. For the adhesive system specimens, before light-curing, all the visible air bubbles trapped in the adhesive bulk were carefully removed using a hypodermic needle [42]. The specimens were light-cured for 20 s at an output irradiance of 600 mW/cm^2^, using a QTH light-curing unit (Optilux 501, Demetron, Danburry, CT, USA) with a light guide with an 11 mm diameter tip. The irradiance of the light-curing unit was continuously checked using a radiometer (model 100, Demetron, Danburry, CT, USA). The specimens with diameters of 15 mm and 10 mm were light-cured in 5 and 3 overlapping sections, respectively, from their top and bottom surfaces. After the first light irradiation, the light guide was moved until the whole surface had been irradiated [9]. For the 6 mm diameter specimens, only a central light irradiation on the top and bottom surfaces was performed. After curing, the specimens were removed from the molds and their peripheries were carefully finished using 1000 grit SiC papers to eliminate flash and irregularities. The laboratory conditions were 23 ± 1 °C and 50 ± 2% relative humidity.

### 2.2. Degree of Conversion DC%

For each specimen dimension, the mold was positioned onto the ATR crystal of the FT-IR spectrometer (Alpha-P/Platinum ATR Module, Bruker Optics GmbH, Ettlingen, Germany) and filled with the respective resin-based restorative material, which was manipulated exactly as described in Section 2.1. The spectra between 1600 and 1800 cm^−1^ were recorded with the spectrometer operating with 40 scans and at a resolution of 4 cm^−1^ (*n* = 5). Afterwards, the materials were light-cured exactly as described in Section 2.1, and the spectra were recorded again using the same parameters. The DC% was calculated from the ratio between the integrated area of the absorption bands of the aliphatic C=C bond (1638 cm^−1^) and that of C=O bond (1720 cm^−1^); it was used as an internal standard and was obtained from the cured and uncured materials, using the following equation:

DC% = [1 − (R polymerized/R unpolymerized)] × 100(1)

where R = integrated area at 1638 cm^−1^/integrated area at 1608 cm^−1^.

### 2.3. Surface Roughness Analysis

Before water sorption and solubility evaluation, all the specimens were analyzed using an atomic force microscope (XE7, Park Systems, Santa Clara, CA, USA). Three areas of 10 μm × 10 μm for each specimen were scanned in contact mode, using a cantilever with a radius of curvature <10 nm, a scan rate of 1 Hz, and a set point of 1.16 nN. The 3D surface topographic was analyzed using XEP software, Version 1.4.0, and the roughness of the surfaces was obtained using the Sa (arithmetical mean height) and Sp (maximum peak height) parameters, using the following equations:

(2) $$Sa=\frac{1}{A}\int_{A}\int \left|Z(x,y)\right|dxdy$$

(3) Sp=max(Z(x,y))

where *x*, *y*, and *Z* are the axes in the three-dimensional space, and *A* is the sample area. 

### 2.4. Water Sorption and Solubility

**Figure 1:**
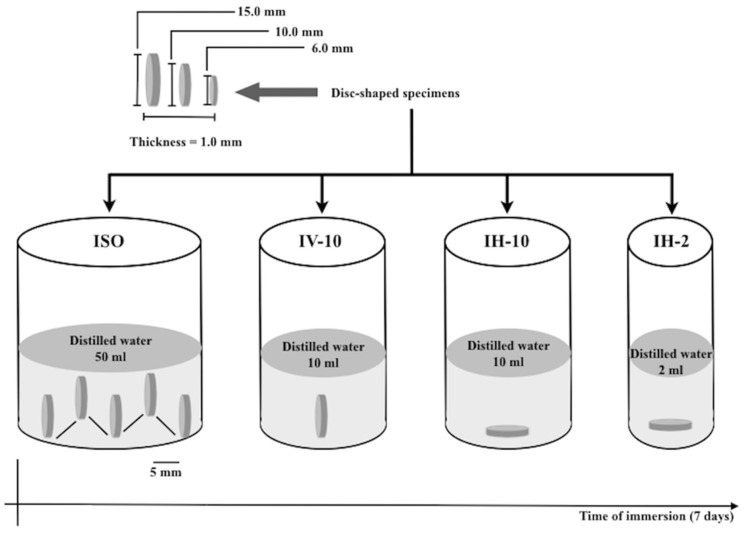
Flow chart for immersion protocols.

Figure 1 shows the flow chart for the immersion protocols evaluated in the present study.

The specimens were placed in individual plastic containers and then stored in a desiccator containing freshly dried silica gel and transferred to an oven at 37 ± 1 °C (Q316B15, Quimis, Rio de Janeiro, RJ, Brazil). After 22 h, the specimens were transferred to another desiccator that was maintained at 23 ± 1 °C for 2 h. The specimens were weighed daily using an analytical balance to an accuracy of 0.01 mg (XP205, Mettler-Toledo, Greifensee, Switzerland) until a constant mass (*m*_1_) was attained (disk mass variation less than 0.1 mg in any 24 h period). After obtaining *m*_1_, the thickness and the diameter of the specimens were measured using a digital caliper (MPI/E-101, Mitutoyo, Kanagawa, Tokyo, Japan) to an accuracy of 0.01 mm. Two measurements of the diameter of each specimen were taken at right angles to each other. The thickness was measured at the center and at four equally spaced points on the specimen circumference. Using the average mean diameter and thickness, the volume (V) of each specimen was calculated in mm^3^.

For each resin-based restorative material, the specimens were immersed in distilled water at 37 ± 1 °C for 7 d according to the following immersion protocols (*n* = 5): ISO (all the specimens were vertically immersed in 50 mL of distilled water, maintaining a 5 mm distance from each other); IV-10 (specimens were individually and vertically immersed in 10 mL of distilled water); IH-10 (specimens were individually and horizontally immersed in 10 mL of distilled water); and IH-2 (specimens were individually and horizontally immersed in 2 mL of distilled water). Racks of nets with rips, glued in the internal wall of the vials, were used for positioning the specimens in the vertical position. The schematic drawing of the immersion protocols is depicted in Figure 1. After 7 d of water immersion, the specimens were removed from the vials, gently wiped with absorbent paper until the visible surface moisture was eliminated, and weighed (*m*_2_). Afterwards, the specimens were reconditioned to a constant mass (*m*_3_) using the same method as for m1.

The water sorption (*W_Sp_*) and the solubility (*W_Sl_*) in µg/mm^3^ were calculated using the following equations:

*W_sp_* = (*m*_2_ − *m*_3_)/*V* (4)

*W_sl_* = (*m*_1_ − *m*_3_)/*V* (5)

### 2.5. Statistical Analysis

The data were analyzed using Statgraphics Centurion XVI software (STATPOINT Technologies, Warrenton, VA, USA). Preliminarily, all the data for each property were submitted to Shapiro–Wilk and Levene tests for the evaluation of the normal distribution of errors and the homoscedasticity of variances, respectively. Based on these results, which proved that all the distributions were normal and homogeneous, the following parametric statistical models were used: the roughness data were submitted to a Student *t* test (Sa × Sp for each resin-based restorative material), the DC% data for each resin-based material were submitted to one-way ANOVA, and the data of the water sorption and solubility for each resin-based restorative material were separately submitted to two-way ANOVA (specimen dimension × water immersion protocol). All the analyses were performed at a significance level of α = 0.05.

## 3. Results

### 3.1. Degree of Conversion DC%

Table 2 shows the results of the DC%. One-way ANOVA showed that for the three resin-based materials, the DC% was not influenced by specimen dimensions (*p* > 0.05).

### 3.2. Roughness

**Figure 2:**
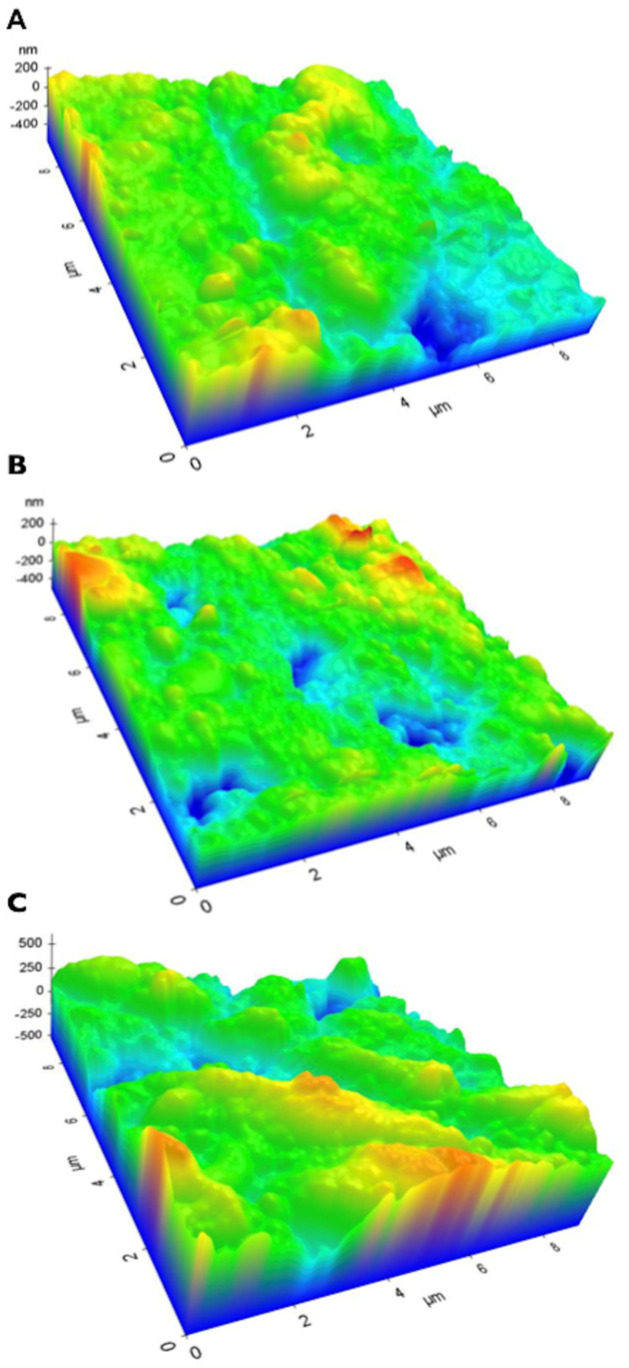
Representative 3D atomic force microscopy images of the three resin-based restorative materials before *W_sp_* and *W_sl_* evaluation: (**A**) resin composite, (**B**) resin cement, (**C**) adhesive system.

**Figure 3:**
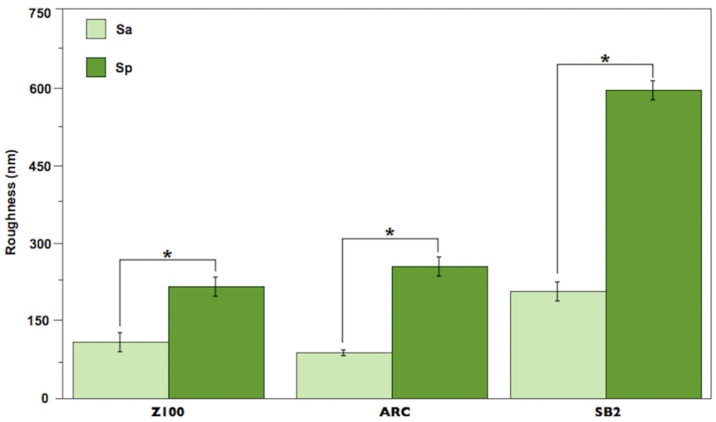
Mean and standard deviation of roughness (nm) for all the resin-based restorative materials. Asterisk indicates statistical difference (*p* < 0.05).

Representative three-dimensional atomic force microscopy images showing the topography of the three resin-based restorative materials are presented in Figure 2: (Figure 2A) resin composite, (Figure 2B) resin cement, (Figure 2C) adhesive system. All the materials showed irregular surfaces, with the presence of craters and peaks of different heights. For the three resin-based restorative materials, Sp was statistically higher than Sa (Figure 3): SB2 (604.2 ± 18.9 nm > 209.1 ± 16.9 nm), Z100 (215.6 ± 18.7 nm > 112.3 ± 19.3 nm), and ARC (255.2 ± 17.3 nm > 93.0 ± 5.8 nm) (*p* < 0.05).

### 3.3. Water Sorption and Solubility

The results of the water sorption and solubility for the three resin-based restorative materials are presented in Table 3 and Table 4, respectively. Two-way ANOVA detected that for each material neither of the properties were influenced by the specimen dimension and water immersion protocol (*p* > 0.05). The values of the water sorption and solubility ranged as follows: Z100 (*W_sp_*: 24.7 µg/mm^3^ to 26.3 µg/mm^3^ and *W_sl_*: 2.0 µg/mm^3^ to 3.2 µg/mm^3^), ARC (*W_sp_*: 19.5 µg/mm^3^ to 22.2 µg/mm^3^ and *W_sl_*: 1.6 µg/mm^3^ to 1.8 µg/mm^3^), and SB2 (*W_sp_*: 96.1 µg/mm^3^ to 101.6 µg/mm^3^ and *W_sl_*: 2.9 µg/mm^3^ to 3.5 µg/mm^3^).

## 4. Discussion

Instead of evaluating the water sorption and the solubility of different resin-based restorative materials, the primary goal of the current study was to investigate how the conditions in which these physical properties are evaluated could interfere with the obtained results. In other words, the aim was to investigate whether the studies of these phenomena have to be conducted strictly according to the parameters established in the ISO 4049 standard [9]. The rationale to this was to verify whether, for a given resin-based restorative material, the results regarding water sorption and solubility obtained in studies using specimens with different dimensions and submitted to different water immersion protocols could be comparable. To the best of our knowledge, this is the first investigation of this issue. 

The monomers that form the organic matrix of the resin-based restorative materials present ester sites (-COO-) that are susceptible to hydrolysis and polar moieties, e.g., –OH, -COOH, -NHCO- and >C=O, and capable of establishing a high level of hydrogen bonds with water molecules [13,45,59,60]. Also, the polymer network formed after their polymerization reaction is heterogeneous, with nano- and micrometer-size voids among polymer chains that favor the inward and outward movement of water molecules as well as their entrapment within the composite bulk. Swelling, plasticization, softening, hydrolysis, release of unreacted monomers, stresses at restoration interface, and lixiviation of fillers and ions are the results of these phenomena; all of them are capable of compromising the physicomechanical properties of the resin-based restorative materials [61,62].

The structure of the polymer network formed after the polymerization of resin-based restorative materials is pivotal for their behavior regarding water sorption and solubility, with the degree of conversion playing a crucial role in this aspect. In the current study, in order to cover their entire volume, the specimens with different dimensions were light-cured with a different number of overlapping sections. Considering the irradiance (600 mW/cm^2^) and the time of irradiation per section (20 s), the specimens received the following total radiant exposure: irradiance × time of irradiation: 15 mm × 1 mm–120 J/cm^2^, 10 mm × 1 mm–72 J/cm^2^, and 6 mm × 1 mm–24 J/cm^2^. Although there is no definite value, according to previous studies [63,64,65] a radiant exposure ranging from 16 to 20 J/cm^2^ is enough to guarantee a suitable degree of conversion for a resin-based material. In the present study, for each resin-based restorative material, the DC% was not influenced by the specimen dimension (Table 2). Although surprising at first glance, this finding may be explained by the radiant exposure to volume ratio received by each specimen. That is, dividing the total radiant exposure per specimen volume, each specimen received: 15 mm × 1 mm–0.7 J/cm^2^, 10 mm × 1 mm–1.0, J/cm^2^, and 6 mm × 1 mm–0.8 J/cm^2^. These numbers allow the claim that the DC% had no influence on the values of water sorption and solubility reached by each specimen.

Neither the dimension of the specimen nor the water immersion protocol influenced the water sorption and solubility values, in agreement with the hypothesis established for the study. According to Sideridou et al. [66], the water sorption phenomenon for resin-based restorative materials is determined by the diffusion coefficient, which is a material constant [67]. By definition, the diffusion coefficient represents the rate of transfer of the diffusing substance across the unit area of a section, divided by the space gradient of concentration in this section [68]. In thin disk-shaped specimens, like those used in the present study, this occurs more often on the parallel surfaces than via the edges. Considering these concepts and the parallel plane area of each specimen used here—15 × 1 (353.25 mm^2^), 10 × 1 (157 mm^2^), and 6 × 1 (56.6 mm^2^)—the absence of statistical significance for the values of water sorption and solubility seems to be unexpected at first glance. However, one explanation for this result is derived from the calculations for water sorption and solubility stipulated by ISO 4049, which are based on changes in density (Equations (3) and (4)). In other words, the water mass gain is proportional not only to the parallel surface areas but also to the specimen volume. Therefore, the parallel surface area to volume ratio is a constant equal to 2 for all the specimens used here: 15 × 1 (353.25 mm^2^/176.6 mm^3^), 10 × 1 (157 mm^2^/78.5 mm^3^) and 6 × 1 (56.6 mm^2^/28.3 mm^3^); this might explain the similarity among the values of water sorption and solubility for each resin-based restorative material.

The specimens of the ISO, IV-10, and IH-10 groups were immersed in 10 mL of water as recommended by ISO 4049. On the other hand, the specimens of group IH-2 were immersed in 2 mL of water only. Taking into account the data in Table 3, it is clear that the worst-case scenario happened with the adhesive system, which showed the highest values of water sorption. Considering the volume of each specimen, 15 × 1 (176.6 mm^3^), 10 × 1 (78.5 mm^3^), and 6 × 1 (28.3 mm^3^), and the values of water sorption for the adhesive system presented in Table 3, it can be calculated that the total mass of water absorbed by the specimens ranged from 2830 µg (group IH-2/specimen 6 × 1 mm) to 17,465.74 µg (group ISO/specimen 15 × 1 mm). As 2 mL and 10 mL correspond to a mass of 2 × 106 µg and 1 × 107 µg of water, respectively, this means that only 0.14% to 0.17% of these masses of water diffused inside the adhesive system of the specimens. Thus, it is plausible to assume that 2 mL of water is much higher than the minimum necessary for saturating the specimens during water sorption evaluation, which seems to be a reasonable explanation for the absence of statistical differences between the specimens immersed in 2 mL or 10 mL of water in the present study. Although it was not the focus of the present study to discuss the behavior of each resin-based material tested here, it is noteworthy that the values of sorption presented by Single Bond 2 (96.1–101.6 μg/mm^3^) were far higher than those presented by Z100 (24.7–26.3 μg/mm^3^) and RelyX ARC (19.5–22.2 μg/mm^3^) (Table 3). These results may be explained by the presence of water and HEMA in the Single Bond composition. HEMA is a low-molecular, highly hydrophilic monomer, that quickly increases its concentration after an initial water volatilization. The consequence of this is a progressive lowering of the vapor pressure of the water in the mixture that jeopardizes the removal of residual water through evaporation [69,70]. Moreover, the presence of Vitrebond copolymer, a polycarboxilic acid that presents a high level of -COOH functional groups in its backbone, could also have contributed to the binding of high a number of water molecules through hydrogen bonding, thereby impacting the water sorption developed by Single Bond 2 [71]. 

**Figure 4:**
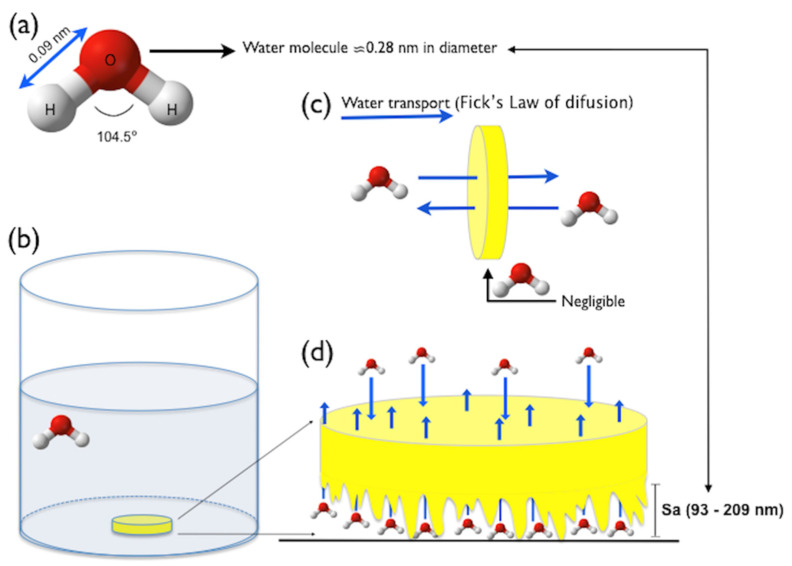
Theoretical model for water diffusion through a specimen horizontally positioned during water sorption and solubility evaluation: (**a**) water molecule, (**b**) specimen horizontally immersed in water, (**c**) water transport according to Fick’s law of diffusion, (**d**) diffusion of water molecules through the rough bottom of the specimen.

The theoretical model depicted in Figure 4 might be used to explain the absence of significant differences in the water sorption and solubility among the specimens that were vertically or horizontally immersed. In Figure 4a, a ball-and-stick model showing that the water molecule has a diameter of ≈ 0.28 nm. In Figure 4b, a specimen of resin-based restorative material horizontally immersed in water suggests that its lower surface is in close contact with the bottom of the vial. Thus, considering Fick’s law of diffusion [67], which establishes that the water uptake in thin disk-shaped specimens occurs principally via parallel disk surfaces because the edge effect is negligible (Figure 4c), it can be supposed that the water diffusion would be impaired in the disk surface theoretically contacting the bottom of the vial. However, taking into account that the surfaces of the three resin-based restorative materials are completely irregular and rough at the nanoscale (Figure 2), and that their nano-roughness ranged from 93 nm to 209 nm (Figure 3), it is reasonable to argue that countless water molecules, which are ≈0.28 nm in diameter, could have easily diffused through the lower, rough side of the specimens (Figure 4d) [72], thereby contributing to the similar water sorption and solubility results obtained here. Moreover, it is important to remember that the nano-roughness schematically represented in Figure 4d is the Sa parameter (Equation (1)), which expresses the average of the absolute values of Z(x,y), being equivalent to the arithmetic mean of the absolute values of roughness when the valleys are changed to peaks. In other words, if we considered the Sp parameter (dark green bars in Figure 3), which expresses the maximum peak height, Zp, on the surface (Equation (2)), the diffusion of water could have been even easier. These aspects confirm that the nano-roughness of the specimens may influence the phenomenon of water transport through the specimens.

In addition to the absence of statistical significance for the specimen dimension and water immersion protocol, it is also noteworthy that, apart from the water sorption of the adhesive system (96.1 µg/mm^3^ to 101.6 µg/mm^3^), all the values of water sorption (19.5 to 26.3 µg/mm^3^) (Table 3) and solubility (1.6 to 3.5 µg/mm^3^) (Table 4) were found to be below the maximum established by the ISO 4049 standard (40 µg/mm^3^ and 7.5 µg/mm^3^, respectively). This aspect corroborates the fact that, for a given resin-based restorative material, different specimen dimensions and water immersion protocols may produce water sorption and solubility results that could be comparable. Some previous studies might reinforce this possibility. Using specimens of 10 × 1 mm, Yap and Wee [32] found water sorption of 29.67 ± 2.10 µg/mm^3^ for Z100. In another study using specimens with 15 × 1 mm [73], the same composite presented water sorption of 28.79 ± 1.16 µg/mm^3^. In the first study, the authors omitted the conditions (volume and position) in which the specimens were immersed in water, while in the second, the author explained that the specimens were vertically immersed in individual containers, without specifying the water volume. Two different studies using specimens with 15 × 1 mm [74] and 12 × 1 mm [75] found water sorptions of 25.51 ± 1.21 µg/mm^3^ and of 23.5 ± 0.6 µg/mm^3^ for Z250. In the first study, the authors did not describe the water immersion conditions, while in the second study the specimens were vertically immersed in water (the volume was not specified). Moreover, after 150 d of water storage, using specimens of 15 × 2 mm, Wei et al. [55] found water sorption of 28.15 µg/mm^3^ for Kalore GC, while Sideridou et al. [66], using specimens of 15 × 1 mm, found a value of 26.15 µg/mm^3^ after 30 d of water storage for the same composite. Even without statistical analyses, it is reasonable to claim that these values of water sorption from different studies could be comparable.

The global analyses of the results obtained in the present study allow interesting comments. First, the absence of the statistical influence of the water immersion protocol on water sorption and solubility can be seen as a desirable aspect by dental scientists because it suggests that the specimens do not need to be vertically positioned in water inside the vial, which is somewhat difficult to perform. Second, the statistical similarity of water sorption and solubility among the specimens with different sizes might represent an economical use of resin-based restorative materials for specimen building, especially when researchers are testing or developing new resin-based materials composed of special and expensive substances [76]. Although this study added interesting aspects regarding the protocol used to evaluate the water sorption and solubility of resin-based restorative materials, limitations such as the use of only distilled water as a storage medium and the absence of long-term evaluation still exist. These aspects should be addressed in future investigations. Although the ISO 4049 standard is a useful guideline for evaluating the water sorption and solubility of resin-based restorative materials, it seems that small variations in specimen dimension and the water immersion protocol in relation to its requirements do not prevent the comparison between the values of these properties obtained in different studies for a given resin-based restorative material.

## Figures and Tables

**Table 1:** Materials used in the present study, with their respective compositions.

Material (Manufacturer)	Type	Composition	Manufacturer
Filtek™ Z100 XT (Z100)	Microhybrid dental composite	Bis-GMA, TEGDMA, 2-benzotriazolyl-4-methyphenol 2, 84.5 wt% Zirconia/Silica	3M ESPE, St. Paul, MN, USA
3M™ RelyX™ ARC (ARC)	Adhesive Resin Cement	BIS-GMA, TEGDMA, pigments, tertiary amine, benzoyl peroxide. Contain inorganic zirconia/silica particles with 67.5 wt% Zirconia/Silica	3M ESPE, St. Paul, MN, USA
Single Bond 2 (SB2)	2-step etch and rinse adhesive	Dimethacrylate resins, 2-hydroxyethyl methacrylate (HEMA), methacrylate-modified copolymer (Vitrebond Copolymer), filler, ethanol, water, initiators	3M ESPE, St. Paul, MN, USA

**Table 2:** Means (±SD) of degree of conversion (DC%) for the three resin-based restorative materials.

15 mm	10 mm	6 mm
	Z100	
66.9 (2.0)	67.3 (2.8)	67.9 (2.7)
	RelyX ARC	
70.9 (2.6)	70.5 (3.0)	68.6 (4.3)
	Single Bond 2	
78.6 (3.2)	77.2 (4.7)	79.6 (2.3)

For each resin-based restorative material there are no statistically significant differences between values of DC% at 5% significance level.

**Table 3:** Means (±SD) of water sorption (μg/mm^3^) for the three resin-based restorative materials.

Groups	15 mm	10 mm	6 mm
		Z100	
ISO	26.0 (1.3)	25.4 (0.5)	25.0 (1.8)
IV-10	26.3 (0.4)	25.7 (0.5)	24.7 (2.2)
IH-10	25.5 (0.4)	26.0 (0.7)	25.7 (1.9)
IH-2	25.8 (0.8)	25.5 (0.6)	24.7 (0.9)
		RelyX ARC	
ISO	19.9 (0.9)	21.0 (1.2)	22.1 (0.5)
IV-10	19.9 (0.7)	21.5 (1.8)	21.6 (2.4)
IH-10	19.6 (0.8)	21.2 (0.5)	22.2 (0.9)
IH-2	19.5 (0.7)	21.5 (0.9)	21.0 (1.5)
		Single Bond 2	
ISO	98.9 (4.5)	100.4 (2.8)	101.6 (0.8)
IV-10	97.1 (5.7)	98.8 (4.0)	101.6 (3.0)
IH-10	96.1 (5.5)	99.5 (3.1)	100.0 (1.5)
IH-2	98.3 (2.7)	101.1 (1.7)	100.0 (1.3)

For each resin-based restorative material there are no statistically significant differences between the values of water sorption (μg/mm^3^) at 5% significance level.

**Table 4:** Means (±SD) of solubility (μg/mm^3^) for the three resin-based restorative materials.

Groups	15 mm	10 mm	6 mm
		Z100	
ISO	2.3 (0.1)	2.5 (0.2)	2.6 (0.5)
IV-10	2.5 (0.5)	2.8 (0.5)	2.3 (0.4)
IH-10	2.0 (0.2)	3.2 (0.7)	2.4 (0.2)
IH-2	2.5 (0.4)	2.5 (1.2)	2.4 (0.3)
		RelyX ARC	
ISO	1.7 (0.2)	1.8 (0.4)	1.7 (0.5)
IV-10	1.7 (0.2)	1.8 (0.1)	1.8 (0.4)
IH-10	1.7 (0.3)	1.8 (0.3)	1.8 (0.4)
IH-2	1.6 (0.3)	1.8 (0.2)	1.8 (0.8)
		Single Bond 2	
ISO	2.9 (1.2)	3.0 (0.3)	3.2 (0.3)
IV-10	3.2 (0.8)	3.0 (0.7)	3.3 (0.0)
IH-10	3.0 (1.4)	3.0 (1.4)	3.4 (0.4)
IH-2	3.1 (0.5)	3.0 (0.3)	3.5 (0.4)

For each resin-based restorative material there are no statistically significant differences between values of solubility (μg/mm^3^) at 5% significance level.

## Data Availability

Data are contained within the article.
